# Professional Dignity

**Published:** 1910-02-19

**Authors:** 


### Professional Dignity.

The age of dignity is past: its place has been taken by the hurry and bustle of an increasingly complex life. As the population of the world grows, it would seem that variety of opinion likewise multiplies, and the old simplicity is lost in the mists of compromise. There is little in our present life that is direct, unless it be the persistent thirst for notoriety. Newton was content to write his great discoveries in a small volume, but the scientific mediocrities of to-day clothe their want of thought in gigantic tomes, replete with graphic methods which are essentially tautologous. But the scientist is daily becoming blinder. The influence of an unprincipled and unrestrained press is permeating the circles of professional men, and the fool rushes into print where the genius was afraid to give utterance even to his thoughts. Little personal "pars" about this or that nonentity seem to have deluded the scientific man into believing that unless he can take the great Public into his confidence he is a mere fly on the wheels of progress, an obstruction to the advance of true learning. And, instead of attempting to popularise science, he tries to make it popular.

Thus it is that we find two professional men, doubtless actuated by highly commendable motives, attacking one another in the pages of a morning paper, while all the world laughs—if it takes any interest at all in the matter. Is it to be supposed that three or four articles in a daily paper will convince anyone of the benefits or evils of vivisection? A man whose suffrage was so easily won would not be worth a position on any side. No good can come of taking the great Public into confidence in this puerile way. Whatever be the merits or demerits of vivisection from the point of view of the man in the street, he will at any rate view with horror this return to the primitive fetishism of the savage, in which animals, to which a Christian Church has denied a soul, are regarded with idolatrous affection. But a discussion in which the one advocate denies the assertions of the other in language which is neither decorous nor classical, is greatly to be deprecated. The vivisectionist cannot do otherwise than regard the anti-vivisectionist as a pestilential fly which spoils the tranquillity of his research. The anti-vivisectionist regards his opponent as a ghoul and a monster. These are matters of opinion. The attitude of each is probably coeval with the egg, and so long as vivisection is not prohibited no harm is probably done by the controversy.

But we cannot view with approval this undignified descent into the public arena where success brings no reward, and failure merely increases the general lack of dignity. Honest as a man may be in his convictions, the proper channel for discussion is not a daily paper in which statements once made, however false they be, are likely to have permanent effect on weak-minded readers. Nor can grave problems such as this is be lightly dismissed with a breath of momentary controversy which redounds to the credit of neither side. Many years ago there used to be an unwritten law against the self-advertisement of the medical profession. It was looked upon with as much respect as that of Holy Orders, and a man was content to sacrifice himself occasionally for the honour of his profession. But nowadays all this is changed. Medicine is increasingly looked upon as a means for making money only, and the old-fashioned altruistic idea of doing some good in one's generation has become less and less followed. Hard though the lot of many a medical man must be, let him live *salva dignitate* rather than at the expense of his dignity.

